# Comparison of fecal and plasma metabolite correlations following nutrition or exercise interventions

**DOI:** 10.1007/s11306-026-02463-z

**Published:** 2026-05-24

**Authors:** Hany Ahmed, Olli Kärkkäinen, Ambrin Farizah Babu, Topi Meuronen, Ville Koistinen, Sophie Leclercq, Camille Amadieu, Quentin Leyrolle, Audrey Neyrinck, Julie Rodriguez, Susanne Csader, Ursula Schwab, Jean-Paul Thissen, Peter Starkel, Philippe De Timary, Sophie Layé, Nathalie Delzenne, Kati Hanhineva

**Affiliations:** 1 Food Sciences Unit, Department of Life Technologies, University of Turku, Turku, Finland; 2 School of Pharmacy, University of Eastern Finland, Kuopio, Finland; 3 School of Medicine, Institute of Public Health and Clinical Nutrition, University of Eastern Finland, Kuopio, Finland; 4 Afekta Technologies Ltd., Microkatu 1, Kuopio, Finland; 5 Laboratory of Nutritional Psychiatry, Institute of Neuroscience, UCLouvain, Brussels, Belgium; 6 Metabolism and Nutrition Research Group, Louvain Drug Research Institute, UCLouvain, Brussels, Belgium; 7 Université de Bordeaux, INRAE, Bordeaux INP, NutriNeuro, Bordeaux, France; 8 Department of Medicine, Endocrinology and Clinical Nutrition, Kuopio University Hospital, North Savo Wellbeing County, Kuopio, Finland; 9 Pole of Endocrinology, Diabetes and Nutrition, Institut de Recherche Expérimentale et clinique, UCLouvain, Université catholique de Louvain, Brussels, Belgium; 10 Cliniques Universitaires Saint Luc, Brussels, Belgium

## Abstract

**Introduction:**

Metabolites are focal players in the host–microbiota crosstalk. Fecal metabolome represents the microbial metabolic output but the links between fecal and circulating metabolites remain unresolved. Deciphering the associations between fecal and plasma metabolomes may benefit the designing of tailored gut microbiota-targeting interventions.

**Objective:**

To study common underlying associations between fecal and plasma metabolites after nutritional and exercise interventions.

**Methods:**

Fecal and plasma samples from three separate interventions of alcohol use disorder (*n* = 44), obese (*n* = 27), or metabolic dysfunction-associated steatotic liver disease (MASLD) participants (*n* = 40) undergoing nutritional or exercise interventions were analyzed using a non-targeted LC-HRMS approach. Annotated features from feces and plasma were subjected to Spearman correlation analysis and fecal–plasma metabolite pairs were compared across studies in baseline, treatment or control groups. Chemical diversity was assessed by over-representation analysis and compound class prediction in the nutritional intervention trials.

**Results:**

The number of nominally significant (*p* < 0.05) metabolite pairs among the alcohol use disorder, obese and MASLD participants were 4250, 5901 and 6981, respectively. Out of the significant metabolite pairs, less than 1% were common among all studies. Assessment of chemical diversity suggested study-specific molecular fingerprints after the nutritional interventions.

**Conclusion:**

Wealth of study- and group-specific correlations were observed between the fecal and plasma metabolites. Lack of significant commonalities between interventions and the divergent chemical landscapes suggests large inter-individual variations in the fecal–plasma metabolite interactions. Distinctive composition of the fecal and plasma metabolomes warrants caution when inferring findings from feces to circulation and further to host health.

**Graphical abstract:**

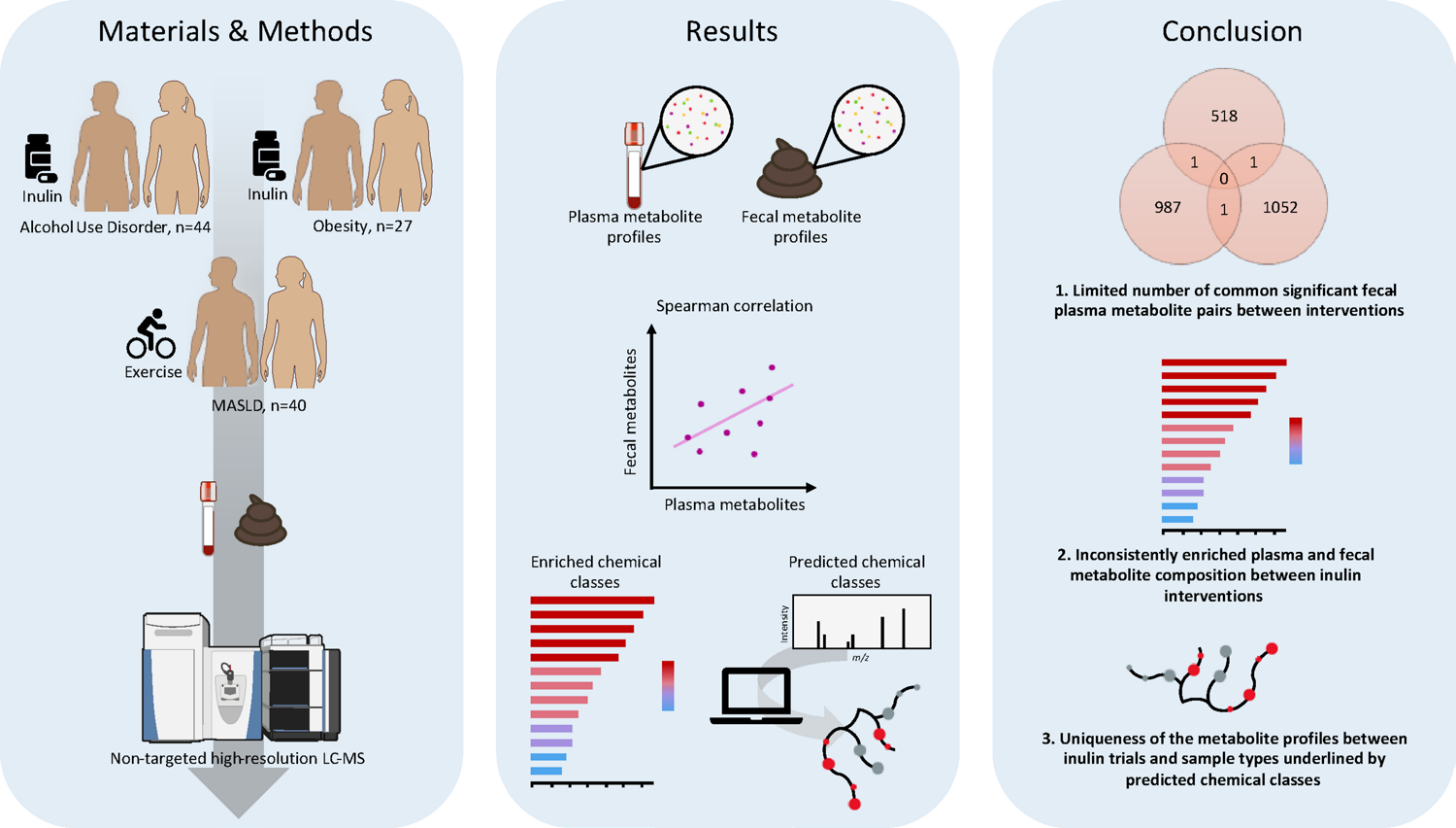

**Supplementary Information:**

The online version contains supplementary material available at 10.1007/s11306-026-02463-z.

## Introduction

The gut microbiota has been linked to, e.g., development, immunity, and metabolic health (Lynch & Pedersen, 2016). Assessment of host metabolomes and gut microbiota composition have revealed the magnitude of gut microbiota–host interplay where metabolites are likely to be among the key crosstalk mediators (Chen et al., 2022; Dekkers et al., 2022). The most representative snapshot of the microbiota’s functional output exists in the gut lumen holding a metabolome that is a mixture of nutritional, host, and microbial metabolites. As the content of the lumen is reachable only by intrusive measures, usage of fecal material as a surrogate matter has been the mainstay in gut microbiota omics studies (Karu et al., 2018). While metabolites of microbial origin are present in plasma and thus can be distributed throughout the host, little is known about the relationship between fecal and blood metabolomes and whether blood metabolite profiles reflect microbial metabolism.

Previous studies on the associations between blood and fecal metabolomes vary on the population size and characteristics, methodology applied and underlying associations revealed (Armstrong et al., 2017; Deng et al., 2023; Ponce-de-Leon et al., 2024). However, these studies have demonstrated that metabolic links between the lumen and circulation exist but their transferability across populations remains uncertain. Discovery of such metabolic links that are consistent and reproducible could be considered as fundamental features of the gut microbiota–host crosstalk and assist in harnessing the potential of gut microbiota for better health.

The metabolic impact of health factors like alcohol use disorder and obesity and lifestyle factors like diet and exercise have been assessed rigorously. Chronic alcohol use and obesity alter liver function that is mirrored by a myriad of abnormalities in amino acid, lipid and steroid metabolism (Cirulli et al., 2019; Voutilainen & Karkkainen, 2019). In metabolic dysfunction-associated steatotic liver disease (MASLD), liver status is gradually worsening and metabolic changes in the circulation are widespread (Tan et al., 2024). The mentioned conditions are generally accompanied by poor diet quality that is reflected by nutrition deficiencies and altered gut microbiota composition and function (Amadieu et al., 2022; Bradley et al., 2023). Moreover, diet and exercise have been shown to induce changes in both endogenous and microbial metabolites but their effect on correlations between the fecal and plasma metabolites have not been compared previously (Bar et al., 2020; Pietzner et al., 2021). Therefore, the purpose of this study was to assess the metabolite correlations between fecal and plasma metabolomes and their stability across different populations. We utilized existing fecal and plasma metabolomic datasets from three distinct clinical studies involving patients with alcohol use disorder participating in 3-week alcohol withdrawal program (herein on the Gut2Brain study), participants with obesity taking part in an 3-month inulin-enriched dietary intervention (herein on the Food4Gut study) and for reasons of data availability, patients with metabolic dysfunction-associated steatotic liver disease participating in a 3-month high-intensity interval training regimen (herein on the BestTreat study). The results describing the metabolic differences observed in the mentioned studies have been previously published (Amadieu et al., 2025; Babu et al., 2022; Leclercq et al., 2024; Leyrolle et al., 2021). In the current study, our aim was to assess the potential persistence by detecting reproducible fecal–plasma metabolite associations within and between the described human intervention trials.

## Methods

### Study samples

#### Gut2Brain study

50 persons with alcohol use disorder, recruited in Brussels, Belgium, were enrolled to the Gut2Brain study which was a randomized, placebo-controlled clinical intervention to study the effects of gradually increasing dietary fiber intake (up to 16 g/day of inulin) on gut microbiota and various psychological and clinical markers over 3 weeks (Amadieu et al., 2022). Fasting blood was collected during the baseline and end visits in the morning at the clinic using EDTA tubes. Blood was immediately centrifuged at 1000*g* for 15 min at + 4 °C and the plasma stored at −80° until use. Fecal samples were collected during the baseline and end visits at the clinic by the participants using a sterile fecal sample collection tube. The sample tube was immediately stored at −20° and transferred to −80 °C within 5 to 10 h of collection. Here, we included as subsample of participants with both plasma and fecal metabolomics data available from the start and end of the intervention. Together, the total number of samples included were *n* = 44 at baseline, *n* = 19 in the placebo and *n* = 17 in the treatment group at the end of the study. All study participants signed informed consent prior to inclusion, and the study was registered in the clinicaltrials.gov registry (identifier: NCT03803709). Details of the Gut2Brain study and clinical characteristics are described in the original publication (Amadieu et al., 2022).

#### Food4Gut study

150 obese participants, recruited in Brussels, Belgium, were enrolled in a multicentric, single-blind, placebo-controlled intervention to study the effects of a 12-week inulin-rich vs. inulin-poor hypocaloric diets on the gut microbiota composition, anthropometric and clinical parameters (Hiel et al., 2020). Participants were randomized to either consume inulin 16 g/day together with an inulin rich diet or placebo (maltodextrin) with inulin poor diet. Fasting blood was collected during the mornings of baseline and end visits in EDTA tubes, immediately centrifuged at 2000*g* for 10 min at + 4 °C and the plasma stored at −80 °C until use. Fecal samples were collected by the participants using a sterile fecal sample collection tube maximum of 24 h prior to baseline or end visits. Participants were instructed to keep the fecal samples at −20 °C and transfer them in cool boxes to the visits where the samples were transferred to −80 °C. Participants with all fecal and plasma metabolomics data available from both timepoints were included in the subset of samples here together making *n* = 27 at baseline, *n* = 14 in the placebo and *n* = 12 in the treatment group at the end of the study. Study setup and clinical characteristics of the participants are detailed in the original publication (Hiel et al., 2020).

#### BestTreat study

49 participants with MASLD, recruited in Kuopio, Finland, were enrolled in a 12-week randomized, controlled exercise intervention study where treatment group followed a twice a week high-intensity interval training regimen while the control group maintained their standard sedentary lifestyle (Babu et al., 2022). Fecal samples were collected at home maximum of 24 h prior to visit, placed on icebox filled with ice bags and brought to the research unit. An anerobic generator bag was placed in the container for the end visit fecal sample. At the research unit, fecal samples were homogenized, aliquoted and stored in −80 °C without any detergents. Fasting blood samples were drawn in EDTA tubes in the morning of baseline and end visits, immediately processed and plasma stored at −80 °C until use. Similar to the other two studies, here we included a subset of participants with all fecal and plasma metabolomics data available from both timepoints. Together, the number of participants were *n* = 40 at baseline, *n* = 20 in the control and *n* = 20 in the treatment group. A detailed explanation of study setup and participant description are available in the original publication (Babu et al., 2022).

### Non-targeted LC-MS metabolomics analysis

#### Metabolite extraction

The extraction protocols for the plasma and fecal samples have been previously described (Babu et al., 2022; Leclercq et al., 2024; Leyrolle et al., 2021). In general, all samples were randomized prior to treatment, thawed on wet ice, mixed with extraction solvent, vortexed, centrifuged and filtered through 0.2 μm PTFE filters. In all studies 100 µL of plasma was mixed with 400 µL of acetonitrile. Gut2Brain fecal samples were mixed with cold water 1:3 (v/w) ratio and diluted with methanol to reach 80% methanol concentration. BestTreat fecal samples were mixed with 80% methanol in a ratio of 500 µL of solvent per 100 mg of fecal material and homogenized using Bead Ruptor 24 Elite homogenizer. Food4Gut fecal samples were mixed with 80% methanol in a ratio of 500 µL of solvent per 100 mg. Study specific QC samples were prepared by combining an aliquot from each sample in a separate vial. Preparations of analytical blanks devoid of sample followed the similar treatment steps as the samples. Samples were stored in −20 °C until analysis.

#### Analytical platforms

The analytical platforms and parameters used to perform data acquisition have been described in the original publications for Gut2Brain (Amadieu et al., 2025; Leclercq S, 2024), BestTreat (Babu et al., 2022) and Food4Gut (Leyrolle et al., 2021) plasma analysis. The Food4Gut fecal sample analysis utilized the same platform and parameters as in the analysis of Gut2Brain fecal material (Amadieu et al., 2025). The same combination of reversed-phase and HILIC separation methods were used for all samples. Instruments were calibrated prior to analysis and recalibration applied across the sample sequence to maintain high mass accuracy (< 2 ppm). Analyses were conducted in ESI + and ESI- modes and data collected in centroid mode. The sequences consisted of blank injections followed by QC injections to equilibrate the instruments and QC injections repeated after every 12 samples. Data-dependent MS/MS scans were applied on QC samples.

#### Peak picking and data processing

MS-DIAL versions 4.24 (BestTreat) and 4.82 (Food4Gut and Gut2Brain) were employed for automated peak picking and alignment(Tsugawa et al., 2015). Following peak picking, the projects were exported as.xlsx files yielding a total of 4 feature tables, ionization modes and chromatographic separations combined, for each matrix within the included studies. For the pre-processing, feature tables were merged into single table and subsequently imported to R statistical software versions 3.6.1 (BestTreat) and 4.0.3 (Food4Gut and Gut2Brain) (R Core Team, 2024). Features were normalized by fitting a feature-wise cubic spline regression to the QC sample peak areas and applying a smoothing parameter to avoid overfitting separately for each analytical batch. Drift correction, detection of low quality signals and missing value imputation were conducted by *notame* R package (Klåvus et al., 2020).

Metabolites were annotated by comparison of chromatographic and mass spectrometric characteristics against an in-house library, publicly available databases such as HMDB (Wishart et al., 2022) and LipidMaps (Conroy et al., 2024) and published literature. In silico fragmentation tools such as MSFINDER (Lai et al., 2018) and SIRIUS (Duhrkop et al., 2019) were utilized for predicting the molecular formula of unknown features. Annotation of metabolites and the level of identification was based on the recommendations given by the Chemical Analysis Working Group (CAWG) Metabolomics Standards Initiative (MSI): 1 = identified based on a reference standard, 2 = putatively annotated based on physicochemical properties or similarity with public spectral libraries, 3 = putatively annotated to a chemical class and 4 = unknown (Sumner et al., 2007). Initially, features were shortlisted for annotation based on group-wise comparisons within studies. All annotated features were cross-checked manually from each dataset by comparison of *m/z* values, retention times and fragmentation spectra between datasets. Features with an average peak area below 100,000 (Orbitrap) or 10,000 (Q-ToF) derived from the sample treatment materials or having over 50% of missing values were excluded from further analysis.

### Statistical analysis

R software version 4.0.3. was used for statistical analysis (R Core Team, 2024). To test the association between annotated fecal and plasma metabolites, the cor.test function was used to calculate Spearman’s correlation coefficients (r_s_) and significance (*p*) without including covariates in the analysis. The *p*-values were adjusted with the Benjamini-Hochberg false discovery rate (*q*-value). Metaboanalyst 6.0 platform was used to perform metabolite set enrichment analysis to analyze significantly altered chemical classes among the annotated metabolites in the inulin trials (Pang et al., 2024). Thresholds for statistical significance were *p*-value and *q*-value below 0.05.

## Results

### Annotated metabolites

**Fig. 1:**
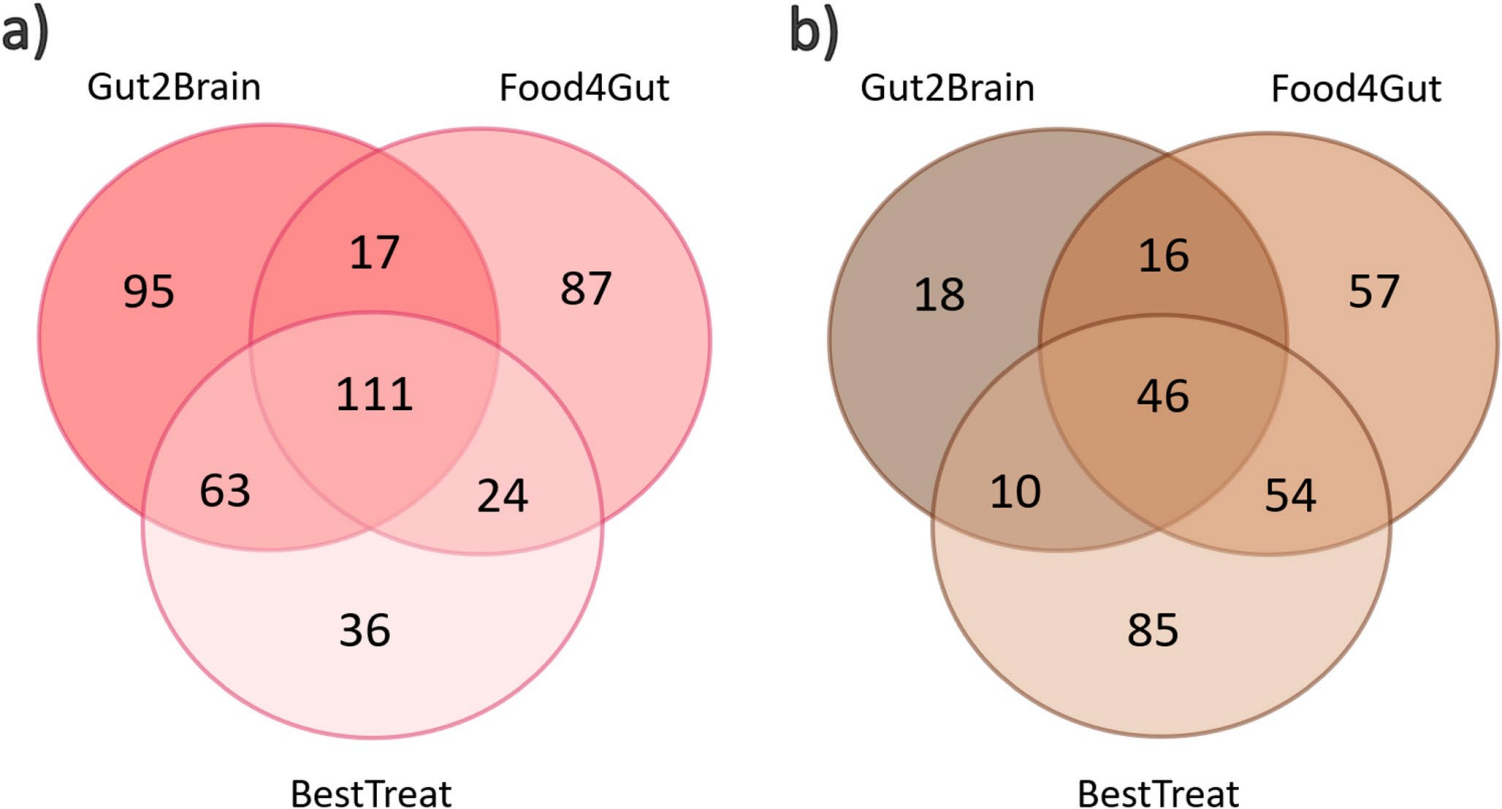
Venn diagrams of annotated metabolites. **A** Number of annotated plasma metabolites in Gut2Brain, Food4Gut and BestTreat studies. **B** Number of annotated fecal metabolites in Gut2Brain, Food4Gut and BestTreat studies

After cross-checking all the annotated metabolites (level 1–3), a total of 111 plasma and 46 fecal metabolites were common across all datasets (Fig. 1). The number of metabolites annotated from the plasma were 286, 239 and 234 in the Gut2Brain, Food4Gut and BestTreat datasets, respectively. The number of annotated fecal metabolites were 90, 173 and 195 from the Gut2Brain, Food4Gut and BestTreat datasets, respectively. Collectively, the plasma metabolome was characterized by metabolites belonging to classes of amino acids, acylcarnitines, fatty acids, (lyso)phosphocholines and steroids including bile acids (Supplementary Table 1.). At the same time, the fecal metabolome was characterized by amino acids, bile acids, dicarboxylic acids and fatty acids.

### Correlations between annotated metabolites within the studies

**Fig. 2:**
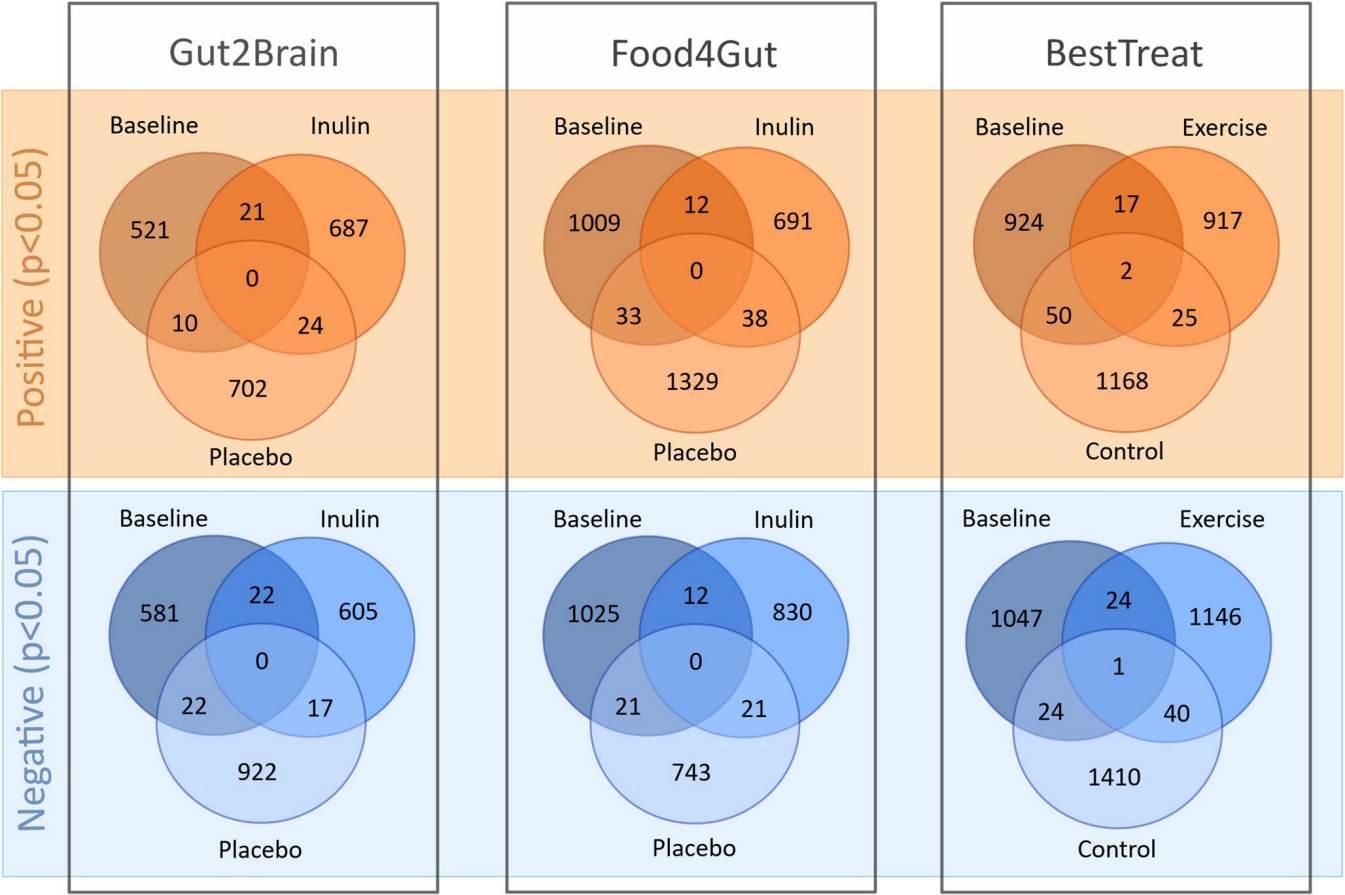
Venn diagrams of the overlapping statistically significant (*p* < 0.05) correlations between fecal and plasma metabolites. Fecal–plasma metabolite pairs were compared between the baseline, treatment and placebo/control groups within the Gut2Brain, Food4Gut and BestTreat studies. Red color indicates correlations with Spearman correlation coefficient values (r_s_)> 0 and blue color coefficient values < 0

The number of significant correlations (*p* < 0.05) within different interventions at baseline or at the end in the individual study groups are summarized in Table 1. None of the correlations remained significant (*q* < 0.05) after adjusting for false discovery rate. The lowest number of significant correlations were observed in the Gut2Brain study with 1177 correlations at the baseline followed by 1376 in the inulin and 1697 in the placebo group at the end of the intervention. In the Food4Gut intervention the number of significant correlations between plasma and fecal metabolites at baseline were 2112 and 1604 in the inulin group or 2185 in the placebo group at the end of the intervention. The greatest number of significant correlations were observed in the BestTreat intervention where 2089 associations were significant at baseline, 2172 in the exercise and 2720 in the control groups at the end of the intervention. Assessment of the persistence of the significant correlations within the studies was done by comparison between the observations at the baseline and at the end of the intervention (Fig. 2, Supplementary Table 2). The Venn diagrams show a similar pattern within all studies – the majority of the significant positive and negative correlations are time- or group-dependent. Regardless of the intervention group, the metabolites forming significant correlations change in the course of time.

**Table 1:** Statistically significant (*p* < 0.05) correlations between plasma and fecal metabolites within the Gut2Brain, Food4Gut and BestTreat studies at baseline and at the end of the intervention

Study	Timepoint	*n*	Sig. correlations (*p* < 0.05)	Positive correlations		Negative correlations	
Gut2Brain	Baseline	44	1177	552	(r_s_ > 0.29)	625	(r_s_ < −0.29)
	End	17 (Inulin)	1376	732	(r_s_ > 0.48)	644	(r_s_ < −0.48)
	End	19 (Placebo)	1697	736	(r_s_ > 0.46)	961	(r_s_ < −0.46)
Food4Gut	Baseline	27	2112	1054	(r_s_ > 0.38)	1058	(r_s_ < −0.38)
	End	12 (Inulin)	1604	741	(r_s_ > 0.58)	863	(r_s_ < −0.64)
	End	14 (Placebo)	2185	1400	(r_s_ > 0.53)	785	(r_s_ < −0.53)
BestTreat	Baseline	40	2089	993	(r_s_ > 0.31)	1096	(r_s_ < −0.31)
	End	20 (Exercise)	2172	961	(r_s_ > 0.44)	1211	(r_s_ < −0.44)
	End	20 (Control)	2720	1245	(r_s_ > 0.44)	1475	(*rs* < −0.44)

r_s_ depicts the threshold of Spearman correlation coefficient for the significant correlations

### Fecal–plasma metabolite correlations between studies

**Fig. 3:**
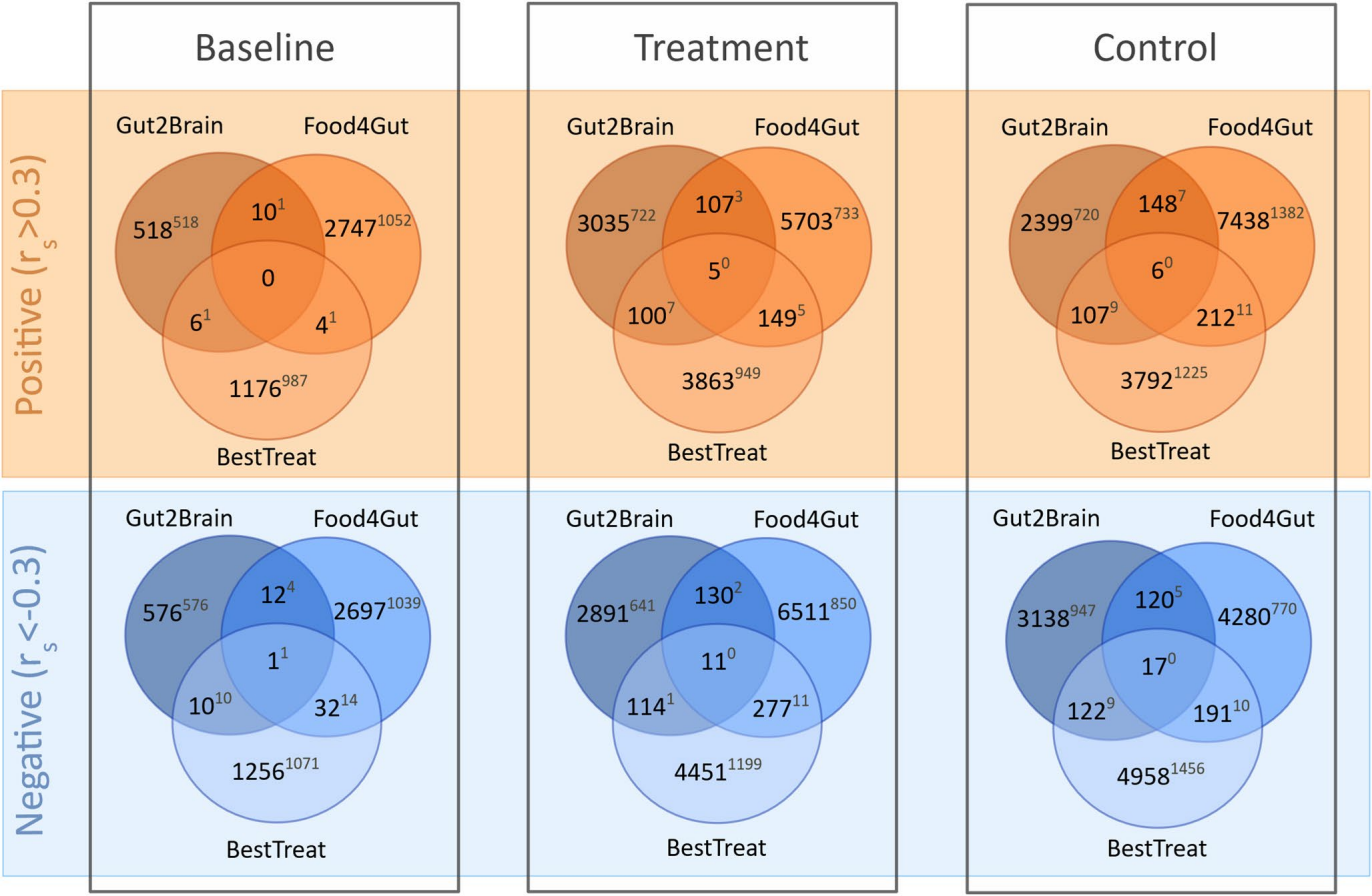
Venn diagrams of correlations between fecal and plasma metabolite profiles with Spearman correlation coefficient (r_s_) of > 0.3 or < −0.3 between the nutritional interventions Gut2Brain and Food4Gut and the exercise intervention BestTreat. Correlations were compared at baseline and after receiving either treatment or placebo/control. Red colors indicate correlations with r_s_ values > 0.3 and blue colors r_s_ values < −0.3. Superscripts denote the number of statistically significant (*p* < 0.05) correlations

Considering the variation in sample sizes, that ranged from up to 44 at the baseline in Gut2Brain down to 12 in the Food4Gut inulin group, correlations with high correlation coefficient (r_s_) may not reach the level of statistical significance in the smallest groups. Hence, we applied a r_s_ threshold when selecting correlations to comparison instead of statistical significance (*p* < 0.05). The Spearman r_s_ threshold of > 0.3 or < −0.3 was selected based on the correlations reaching the level of statistical significance in the group with highest sample size, i.e. Gut2Brain baseline with *n* of 44. Venn diagrams of the correlations with Spearman r_s_ of > 0.3 or < −0.3 between the studies are displayed in Fig. 3. Despite several thousands of metabolite pairs subjected to comparison, the total number of shared correlations between interventions were 1 at baseline, 16 in the treatment and 23 in the placebo/control groups. The relative proportion of shared correlations between two or more studies did not exceed 4% in the baseline, treatment or placebo/control groups. In the treatment groups there was no single metabolite driving the common correlations although metabolites belonging to phosphocholines, particularly lysophosphatidylcholines, characterized the negative correlations. In the placebo/control groups however, plasma lysophosphatidylcholines with fecal amino acid derivatives urocanic and glutaric acids were driving the negative associations while the positive correlations were characterized by plasma amino acids and fecal lysophosphatidylcholines.

The number of significant correlations, as indicated by the superscripts in Fig. 3, was far lower than the total number of metabolite pairs compared. A single negative correlation at the baseline between plasma O-palmitoleoylcarnitine and fecal trigonelline was common across the three studies. Overall, the proportion of significant correlations shared between two or more studies did not exceed 1% regardless of evaluating the baseline, treatment or placebo/control groups. The highest numbers of shared significant correlations were demonstrated with the placebo/control groups; 21 between the Food4Gut and BestTreat groups or 18 between the Gut2Brain and BestTreat groups. Within these shared associations, positive correlations were largely characterized by plasma and fecal amino acids and their derivatives while negative correlations by lipids such as phosphocholines, fatty acids and sphingomyelins.

### Chemical diversity of the plasma and fecal metabolites following inulin supplementation

**Fig. 4:**
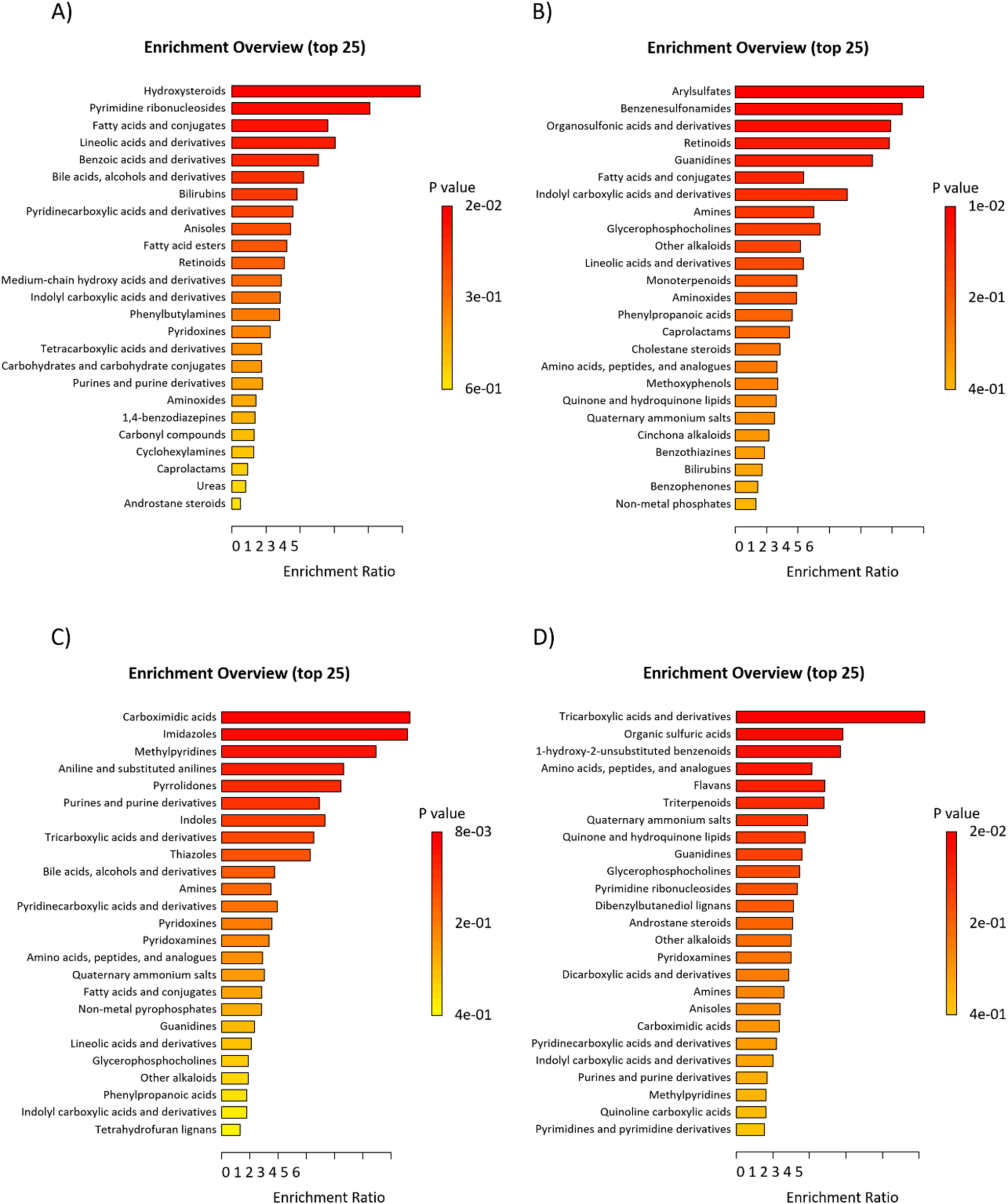
Top 25 enriched chemical classes within all annotated plasma and fecal metabolites between the 3-week Gut2Brain and 12-week Food4Gut inulin intervention treatment and placebo groups. **A** Enriched plasma metabolite chemical classes after 3-week intervention. **B** Enriched plasma metabolite chemical classes after 12-week intervention. **C** Enriched fecal metabolite chemical classes after 3-week intervention. **D** Enriched fecal metabolite chemical classes after 12-week intervention. Enrichment ratios and statistical values derived from Metaboanalyst 6.0 using the quantitative enrichment analysis algorithm

Given the absence of notable shared correlations, we conducted a metabolite set enrichment analysis on the annotated metabolites to seek any shared patterns on the chemical class level in the inulin interventions. Figure 4 displays the enrichment overviews for the top 25 chemical classes enriched in each dataset separately. Figure 4A and B display the enriched plasma chemical classes after the 3-week (Gut2Brain) and 12-week (Food4Gut) intake, respectively. None of them remained significant after adjusting but several classes were nominally significant (*p* < 0.05). Significant classes the Gut2Brain plasma were hydroxysteroids and pyrimidine ribonucleosides while arylsulfates, benzenesulfonamides, organosulfonic acids and derivatives, retinoids, guanidines, fatty acids and conjugates and indolyl carboxylic acids and derivatives in the Food4Gut plasma. As with plasma, only nominally significant class enrichment was observed in feces although none were shared with plasma. Enriched chemical classes in the Gut2Brain fecal metabolome were carboximidic acids, imidazoles, methylpyridines, aniline and substituted anilines, pyrrolidones, and purines and purine derivatives (Fig. 4C) while in the Food4Gut fecal metabolome only the class of tricarboxylic acids and derivatives was nominally significant (Fig. 4D). Together, the enrichment analysis further indicated heterogenous and chemically diverse plasma and fecal metabolomes following varying length inulin supplementation.

**Fig. 5:**
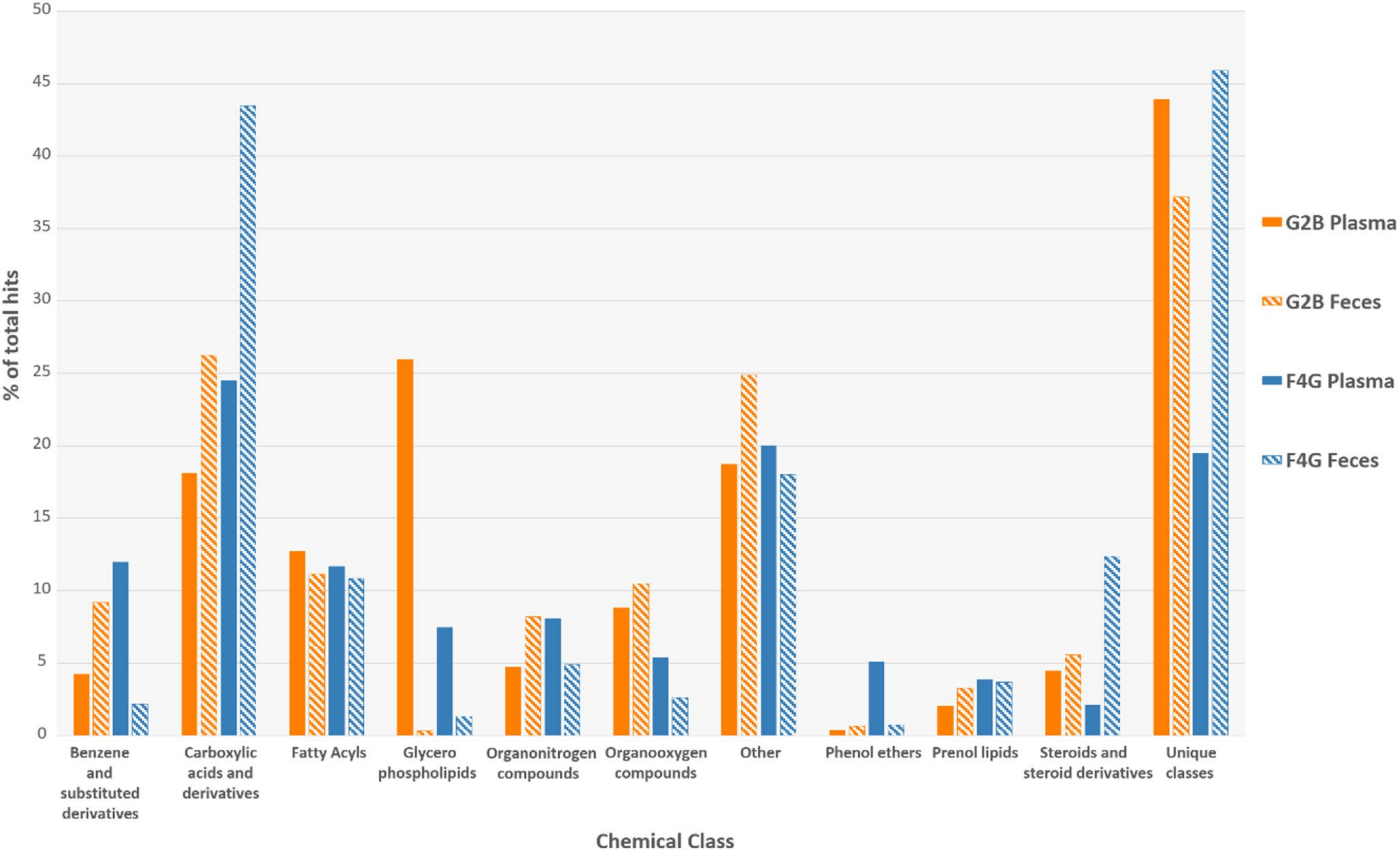
Predicted plasma and fecal molecular fingerprints between Gut2Brain (G2B, orange) and Food4Gut (F4G, blue) studies utilizing acquired reversed-phase separated positive-ionization MS/MS data. Bars indicate relative distributions of top 10 commonly assigned ClassyFire chemical classes and sum of unique classes from plasma and fecal metabolomics data using Sirius 5.8.5 and integrated CSI: FingerID

However, simply describing the metabolome by the annotated metabolites does not necessarily capture the whole complexity of the chemical landscape. Hence, we also utilized the acquired MS/MS (reversed-phase, positive ionization) from the Gut2Brain and Food4Gut datasets to systematically predict and assign ClassyFire chemical class from the fragmentation spectra (Duhrkop et al., 2021). This computational approach indicated that metabolites belonging to classes of carboxylic acid and derivatives, fatty acyls, glycerophospholipids and organooxygen compounds characterized the plasma metabolome (Fig. 5, Supplementary Table 3). Instead, fecal metabolome was characterized by carboxylic acids and derivatives, fatty acyls, steroids and steroid derivatives and organonitrogen compounds. The heterogeneity of the metabolomes was underlined by roughly 20–45% of the assigned classes being unique to each dataset.

## Discussion

The aim of this work was to investigate potential underlying commonalities between fecal and plasma metabolites using untargeted metabolomics data collected from subsets of three separate clinical intervention studies. While such comparison may not offer insight on the complexity of the relationships between individual metabolites, it can reveal universal fecal–plasma metabolite links independent of host health or lifestyle factors. Correlation analysis revealed hundreds of time- and group-dependent correlations between fecal–plasma metabolites within interventions but only a few correlations were shared between interventions regardless of applying either statistical or correlation coefficient thresholds. Moreover, the common effect of inulin supplementation on the fecal and plasma metabolomes was inconclusive as indicated by the correlations and the analysis of the chemical class diversity and metabolite enrichment. Hence, the findings do not provide evidence about consistently observed fecal–plasma metabolite correlations across treatments or populations but rather suggest they are dynamic, modulated by intra-individual factors, such as overall health status, gut microbiota and dietary factors. Such factors should be carefully considered or matched when combining data from different populations.

The growing number of annotated metabolites was in direct relationship with the number of significant correlations between fecal and plasma metabolites reaching several hundred within groups. However, in our analysis, correlations were rendered nonsignificant after adjusting for false discovery rate. In previous studies where correlation analysis has shown significant correlation after correction for multiple testing with several hundreds of fecal and plasma metabolites, metabolomics data has been obtained from over 1,000 individuals. In a study with 132 paired metabolites from over 1,000 middle-aged and elderly adults Spearman correlation analysis adjusted for age, sex, and BMI showed low phenotypic r_s_ of 0.05 ± 0.12 with eight metabolite pairs having r_s_ > 0.3 and a *q*-value < 0.05 (Deng et al., 2023). In an even larger cohort of 1,370 participants with metabolic syndrome, two correlations had *q*-value < 0.05 displaying a correlation coefficient of over 0.20 (Ponce-de-Leon et al., 2024). Moreover, the studies utilized targeted metabolomics data which could limit the number of correlated metabolites and thus require less stringent correction for multiple testing. Thus, the findings suggest that even larger study samples are required to detect significant adjusted correlations that demonstrate weak coefficients if using nontargeted metabolomics data.

In our analysis, nominally significant correlations had moderate r_s_ and applying either nominal significance or r_s_ threshold did not reveal substantial commonalities between the included study datasets. The study and group specificity of the observed correlations suggest that host health status is an important determinant concerning the correlation patterns. For instance, in patients with myalgic encephalomyelitis or chronic fatigue syndrome, numerous correlations were observed between serum metabolites of gluconeogenesis or purine synthesis pathways and fecal amino acids and short-chain fatty acids, but the patterns were different between case and control groups (Armstrong et al., 2017). In patients with colorectal cancer or colorectal adenoma, between 27 paired plasma and fecal metabolites, several significant correlations were shared between study groups (Sun et al., 2024). However, fecal and plasma metabolomes had few overlapping differential metabolites and inconsistently altered metabolic pathways. In women with or without HIV, fecal–plasma metabolite correlations had a median r_s_ of 0.04 in women HIV patients compared to median r_s_ of 0.13 in HIV-free women (Jia et al., 2025). Interestingly, associations between metabolites and type 2 diabetes were also weaker in the patient group. Metabolites may also link specific disease markers as network analysis linked several fecal organic and bile acids to serum amino acids and their derivatives further associated with multiple cardiometabolic traits (Ling et al., 2024). Metabolite and disease-specific associations were also observed when studying how fecal and plasma metabolites were associated with type 2 diabetes, obesity and other cardiometabolic diseases. Hence, metabolite correlations may reveal underlying metabolic regulation networks that are unique to a given health condition and intervention.

Despite the vast number of correlations between fecal and plasma metabolites we and others have observed, the lack of consistency among them is surprising. This may be explained by the pools of studied metabolites as determinants for plasma metabolites include diet, chemical exposure, clinical data, microbiome and genetics all shaping the metabolome in a unique manner (Bar et al., 2020; Chen et al., 2022; Pietzner et al., 2021). In contrast to plasma, the gut microbiome explains almost 70% of the fecal metabolome (Deng et al., 2023; Tang et al., 2019; Zhao et al., 2022; Zierer et al., 2018). However, regardless of the variable determinants of plasma metabolome and unique microbiome compositions, studies have shown a proportion of the metabolome to be consistently detected in industrialized populations (Ghosh et al., 2024; Haffner et al., 2022). In our analysis, roughly 50% of plasma metabolites and roughly 45% of fecal metabolites were detected in at least two datasets being in accordance with earlier work. As phosphocholines and amino acids and their derivatives made up a large proportion of the overlapping metabolites, they characterized the common correlations detected between two groups or studies.

It is intriguing to speculate to what extent does the individual properties of gut microbiota shape the fecal–plasma metabolite correlation although no microbial data was included in this analysis. The studies included populations with clinical conditions associated with distinct compositional changes in the gut microbiota and utilized treatments with microbiota-altering effects. Medications like metformin or antidepressants relevant to the studied populations have also metabolism or gut microbiota modulating properties (Bhattacharyya et al., 2019; Hiel et al., 2020). As inulin induces specific changes to genera Bifidobacterium, Lactobacillus and Bacteroides, a characteristic metabolic shift reflected in the fecal–plasma metabolite correlations could be expected (Le Bastard et al., 2020). However, among the inulin-treated groups, only 5 nominally significant correlations were observed and applying the Spearman r_s_ threshold of > 0.3 or < − 0.3 increased the number to 237. While preclinical models have shown that inulin supplementation alters the plasma metabolome (Li et al., 2022; Nakajima et al., 2022), clinical trials demonstrated less pronounced and indecisive effects on fecal and plasma metabolomes. In inulin-supplemented healthy adults the fecal and plasma volatile metabolite profile remained unchanged over 4 weeks (Vandeputte et al., 2017) and extension to 6 weeks did not change the outcome in overweight/obese individuals (Chambers et al., 2019). Yet, a 12-week supplementation of inulin/oligofructose 50/50 mix in women with obesity significantly decreased fecal total short-chain fatty acids but did not modify serum or urinary metabolome (Salazar et al., 2015). As such, the differences in the metabolic fingerprints exhibited by inulin intake may derive from the temporal differences and subsequent phase of metabolic adaptation.

Our work has some limitations worth discussing in more detail. Despite applying a non-targeted metabolomics approach, the choice of analytical setup and applied metabolite extraction steps affect the coverage of metabolites. Here, we report mainly non-volatile metabolites although fecal matter is rich in volatile compounds such as esters, alcohols and ketones (Karu et al., 2018). To improve coverage, combining several extraction techniques and instrument platforms would extend the current overrepresentation of polar metabolites to other classes. The variation in the number of matched annotations across studies may result from methodological factors such as extraction procedure and mass spectrometry analysis. While the extraction solvents were uniform, the steps applied in collecting fecal material and extracting fecal metabolites varied between the studies. Several analytical platforms were applied for data acquisition on different occasions and while carefully maintained, the effects of analytical variability cannot be completely excluded. Regardless of the hundreds of annotated metabolites, the number of participants across the studies was relatively low that was reflected in the statistical power. Additionally, the lack of overlapping metabolites and correlations may rise partially from the heterogeneity of the clinical populations, intervention protocols and duration instead of solely attributed to disease biology. Finally, the absence of compositional data of the gut microbiota precludes detailed analysis of the gut–host metabolic networks linking fecal metabolites to their plasma counterparts. On the other hand, the strengths of this work include similar instrumental parameters, chromatographic separation methods, data preprocessing and metabolite annotation steps applied across the different studies. Moreover, to increase coverage and common metabolites, all annotated metabolites were manually cross-checked between different datasets. However, it should be noted that the unannotated features may also contain potential commonalities.

As we show here, the fecal–plasma metabolite associations are inconsistent due to factors including, but not limited to, inter-individual variability, sample collection and cohort characteristics. While both blood and fecal metabolomic profiling can be separately used for the assessment of dietary and health biomarkers (Karu et al., 2018; Kortesniemi et al., 2023), there seems to be limited additional benefit in studying the associations between these metabolic pools. Currently, the reports of fecal–plasma metabolite associations are sporadic and represent heterogenic clinical populations as in our analysis. The common theme is that these associations are observed in isolation and present in a time-specific manner. To verify that there truly is no ‘core’ association network between the fecal and plasma metabolomes, similar comparisons should be made with larger studies utilizing the same analytical procedures from the sample collection all the way to metabolite annotation. The development of sampling techniques capable of reaching the luminal metabolome provides a better representation of the intestinal community and function than relying solely on fecal material that is essentially a waste product (Folz et al., 2023; Shalon et al., 2023). Integrating intestinal metabolomics and metagenomics data is essential for understanding the metabolic regulation taking place within the gut and between the gut and the host. Moreover, the uptake and metabolism of the luminal metabolites by the intestinal cells or liver via portal vein limits the translocation to circulation or excretion in feces (Koh et al., 2016). In this context, the integration of liver transcriptomics and proteomics provides insights into the gut–liver axis as a regulator of gut-originating signaling.

In conclusion, our findings on numerous group and study-dependent correlations replicated previous isolated reports of their presence but question their comparability between populations. Based on ours and others’ results, host health, nutrition, environmental factors, physical activity and gut microbiota composition shape a unique fecal–plasma metabolic correlation network that is not reproducible across studies. As the correlations changed over time, longitudinal metabolomics studies including both healthy and diseased individuals are paramount for assessing the inter- and intra-individual stability of fecal and plasma metabolic crosstalk. Utilization of validation cohorts that have similar clinical profiles is necessary to account for potential environmental effects and for the influence of metabolic disorders on host biochemistry. This also underscores the importance of including a control group when studying clinical populations. We also showed that circulating metabolome is a poor representation of the fecal metabolome, and that fecal metabolome should not be regarded as a definitive readout of the gut microbiota’s metabolic activity with health relevance. Hence, the influence of the metabolic site on correlations should be carefully considered and avoid extending findings to other metabolic pools without separate assessments.

## Supplementary Information

Below is the link to the electronic supplementary material.

Supplementary file1 (XLSX 123 KB)

Supplementary file2 (XLSX 11817 KB)

Supplementary file3 (XLSX 499 KB)

## Data Availability

Data for the results is provided within supplementary information. Additional spectral data for this study are available from the corresponding author upon reasonable request.
